# Identification and validation of prognostic autophagy-related genes associated with immune microenvironment in human gastric cancer

**DOI:** 10.18632/aging.204313

**Published:** 2022-09-28

**Authors:** Ruyue Tian, Ya Sun, Xuedi Han, Jiajun Wang, Hongli Gu, Wenhai Wang, Lei Liang

**Affiliations:** 1 Department of Ultrasound, Aero Space Central Hospital, Beijing 100050, China; 2 Department of Gastroenterology, Beijing Friendship Hospital, Capital Medical University, National Clinical Research Center for Digestive Disease, Beijing Digestive Disease Center, Beijing Key Laboratory for Precancerous Lesion of Digestive Disease, Beijing 100050, China

**Keywords:** gastric cancer, autophagy-related genes, cancer immunity, prognosis, The Cancer Genome Atlas

## Abstract

Autophagy-related genes (ATGs) play critical roles in tumorigenesis and progression in gastric cancer (GC). The present study aimed to identify immune-based prognostic ATGs and verify their functions in tumor immune microenvironment (TIME) in GC. Macrophage infiltration was found to negatively correlate with prognosis in GC patients. After stratifying by infiltration levels of macrophages, we screened The Cancer Genome Atlas and Human Autophagy Database to identify the differentially expressed ATGs (DE-ATGs). Of 1,433 differentially expressed genes between the two groups, seven genes qualified as DE-ATGs. Of these, *CXCR4*, *DLC1*, and *MAP1LC3C*, exhibited strong prognostic prediction ability in Kaplan-Meier survival–log-rank test. High expression of these genes correlated with increased occurrence of advanced grade 3 tumors and poor prognoses. Furthermore, GSEA indicated that they were significantly associated with oncogenic and immune-related pathways. The comprehensive evaluation of TIME via GEPIA, ESTIMATE, CIBERSORT, and TIMER suggested that the three DE-ATGs were closely associated with immune condition, both in terms of immune cells and immune scores. Thus, the outcome of this study may aid in better understanding of the ATGs and their interaction with the immune microenvironment, which would allow the development of novel inhibitors, personalized treatment, and immunotherapy in gastric cancer.

## INTRODUCTION

Gastric cancer (GC) is a common malignancy and is the third leading cause of cancer-related deaths worldwide [1]. The poor survival in patients with GC is primarily due to late diagnosis and suboptimal therapies. The development of immunotherapy has helped improve outcome in patients with GC [2, 3]. However, majority of the patients with GC fail to respond to immunotherapy, while the initial responders may develop resistance to the treatment [4]. Genetic changes play a critical role in immune evasion and suppression in the tumor microenvironment (TME) that may further regulate the occurrence and development of tumors [4, 5]. Therefore, combining targeted gene therapy with immunotherapy may enhance the treatment effect and overcome resistance to immunotherapy in GC.

Autophagy, similar to a “double-edged sword,” plays a dual role in inhibiting and promoting tumor formation and resistance to treatment [6, 7]. Studies suggest that autophagy can modulate immune responses by influencing immune cells and release of cytokines in the TME [8]. Additionally, the fundamental effects of autophagy on tumor progression and immune responses are mediated by autophagy-related genes (ATGs) [9]. ATGs perform widespread physiological functions in autophagy and other biological pathways, and thus, may provide us with novel therapeutic targets in GC [9, 10]. Studies have demonstrated that combining therapy targeting ATGs (induction or inhibition of autophagy) with immunotherapy may enhance the antitumor effects of immunotherapy and overcome immune resistance [8, 11].

The macrophages function as a connection between autophagy and immunity [11, 12]. As a major component of the TME, tumor-associated macrophages closely resemble the M2 macrophages that are related to immunosuppression and tumor progression in GC [13]. Moreover, using Tumor Immune Estimation Resource (TIMER) (https://cistrome.shinyapps.io/timer/), we found that macrophage infiltration was significantly negatively correlated with patient prognosis in GC, and patients with GC with high macrophage infiltration had poor survival outcome. Additionally, it is possible to screen prognostic ATGs related to GC immunity based on the infiltration of macrophages.

Here, using the Cancer Genome Atlas (TCGA) datasets, we performed differential expression analysis and survival analysis with ATGs related to GC immunity in patients stratified by macrophage infiltration levels. We explored the underlying biological pathways of ATGs and their clinical utility as prognostic signatures. Furthermore, we verified correlation between prognostic ATGs and the tumor immune microenvironment (TIME). We identified potential prognostic targets that may provide a foundation for subsequent immune-related work, such as enhancing antitumor effects of immunotherapy or selecting patients who may benefit from the treatment.

## RESULTS

### Identification of differentially expressed autophagy-related genes (DE-ATGs) related to GC immunity

**Figure 1:**
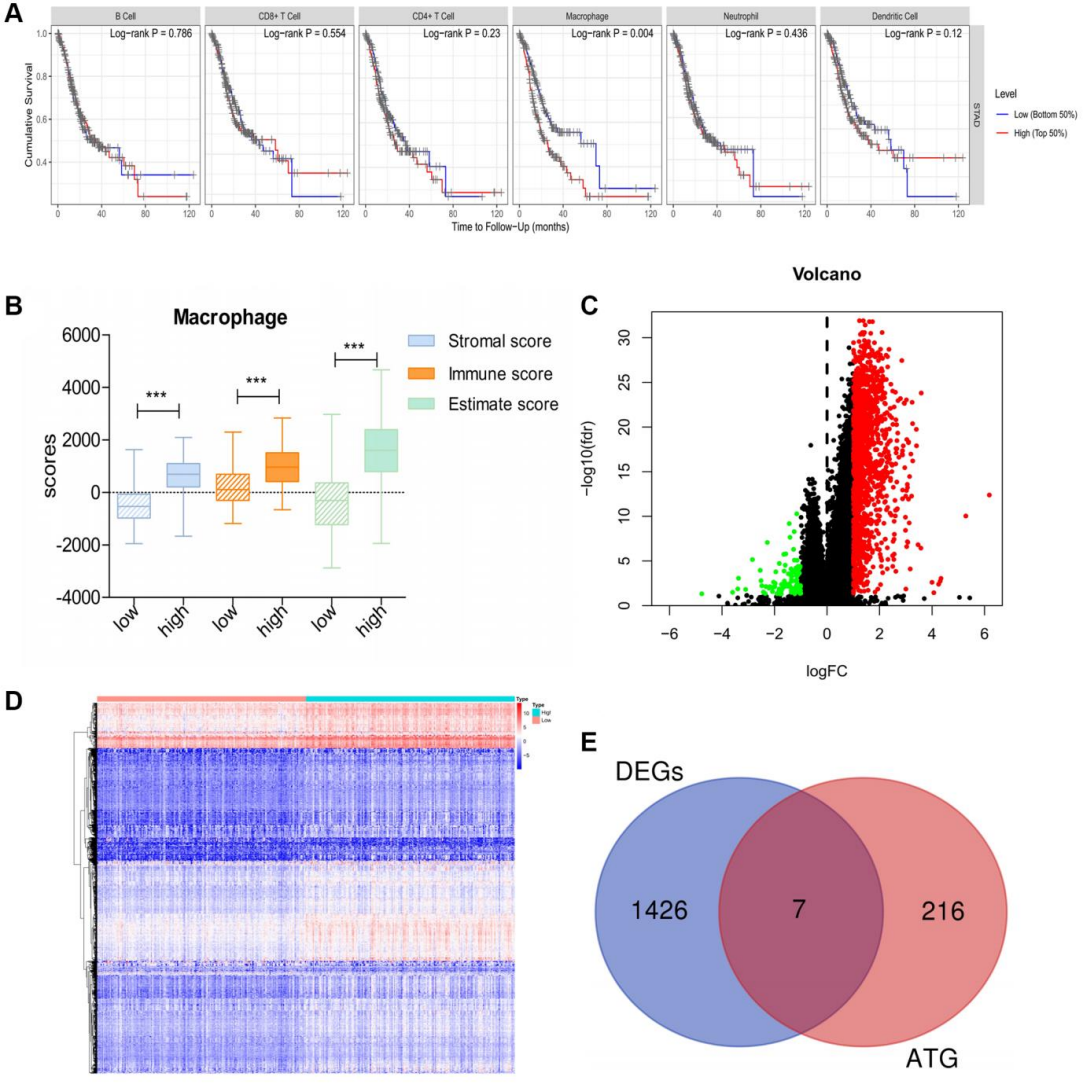
**DE-ATGs related to GC immunity.** (**A**) Macrophage infiltration is significantly negatively correlated with prognosis in patients with GC. (**B**) Distribution of ESTIMATE scores, immune scores, and stromal scores between high- and low-macrophage infiltration groups. (**C**, **D**) Volcano plot and Heatmap of the DEGs between high- and low-macrophage infiltration groups (Upregulated genes are indicated in red dots; downregulated genes are indicated in green dots). (**E**) Venn diagram analysis of DE-ATGs between DEGs and ATGs.

First, using TIMER, we investigated the correlation between overall survival (OS) and abundance of the six types of immune cells (B cells, CD4+ T cells, CD8+ T cells, neutrophils, macrophages, and dendritic cells). The analysis showed that macrophage infiltration was significantly negatively correlated with prognosis in patients with GC (Figure 1A). Based on the median macrophage infiltration levels, patients were segregated into high-infiltration and low-infiltration groups. Patients in the high-infiltration group had higher ESTIMATE scores, immune scores and stromal scores than those in low-infiltration group, indicating that macrophage infiltration levels could accurately reflect the TME (Figure 1B). Next, we performed gene expression data analysis in GC patient samples with variable macrophage infiltration levels, and identified 1,433 differentially expressed genes (DEGs). Of these, 1,337 genes were significantly upregulated and 96 were downregulated in the high-infiltration group than in low-infiltration group (Figure 1C, 1D). Furthermore, seven DEGs were screened as DE-ATGs related to GC immunity (Figure 1E).

### Prognostic significance of DE-ATGs in GC

**Figure 2:**
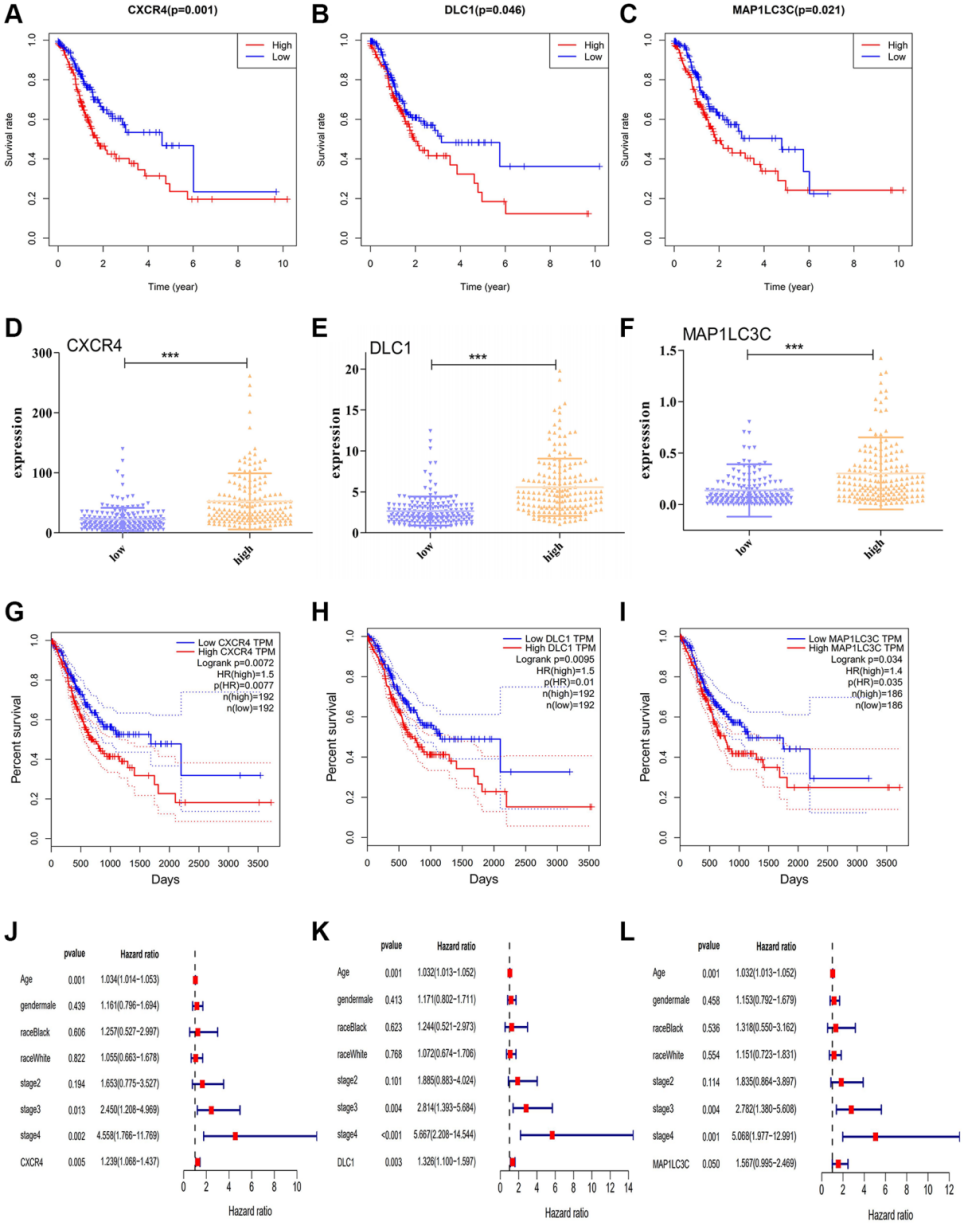
**Prognostic significance of DE-ATGs in GC.** (**A**–**C**) Kaplan-Meier curves for three prognostic DE-ATGs (*CXCR4*, *DLC1*, and *MAP1LC3C*) related to immunity in patients with GC (*P* < 0.05). (**D**–**F**) The expression level of *CXCR4*, *DLC1*, and *MAP1LC3C* in high- and low-macrophage infiltration groups. (**G**–**I**) GEPIA-based validation that DE-ATGs (*CXCR4*, *DLC1*, and *MAP1LC3C*) are effective prognostic indicators and risk factors for GC. (**J**–**L**) Tree diagram of a multivariate regression analysis for *CXCR4*, *DLC1*, and *MAP1LC3C* with other clinical variables.

To assess the predictive significance of the DE-ATGs, we performed Kaplan-Meier survival analysis for the seven DE-ATGs related to GC immunity. The analysis identified three genes as prognostic factors in patients with GC, and that patients with low expression of *CXCR4*, *DLC1*, and *MAP1LC3C*, (*p* = 0.001, *p* = 0.046, and *p* = 0.021) had better prognoses than those with high expression (Figure 2A–2C). The expression levels of *CXCR4*, *DLC1*, and *MAP1LC3C* were significantly different in the high-infiltration and low-infiltration groups (Figure 2D–2F). Moreover, all three genes (*CXCR4*, *DLC1*, *MAP1LC3C*) showed significant prognostic capability in the GEPIA analysis (*p* < 0.05; hazard ratio (HR) > 1; Figure 2G–2I). Furthermore, the multivariate regression Cox analysis suggests that all three genes (CXCR4, DLC1, MAP1LC3C), age and tumor stage are important factors that correlate with survival outcome in GC patients (*p* < 0.05; hazard ratio (HR) > 1; Figure 2J–2L). And, both these analyses demonstrate the effective prognostic prediction by CXCR4, DLC1 and MAP1LC3C.

### Clinical utility of prognostic DE-ATGs (CXCR4, DLC1, and MAP1LC3C) in patients with GC

**Figure 3:**
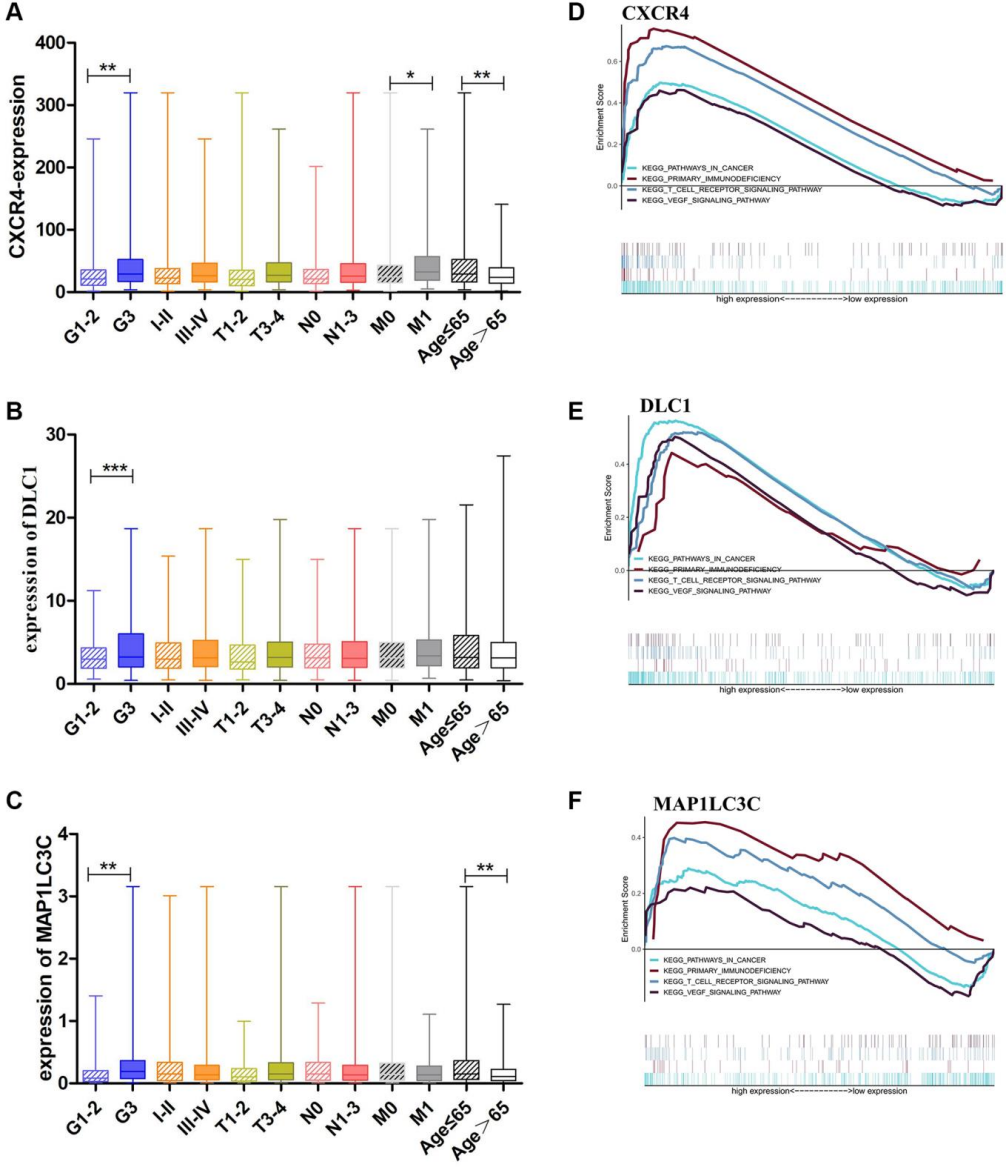
**Clinical evaluation and identification of signaling pathways associated with *CXCR4*/*DLC1*/*MAP1LC3C*.** (**A**, **B**) Expression of *CXCR4*/*DLC1*/*MAP1LC3C* in different grade stage, clinical stage, T stage and age groups in patients with GC. (**C**–**F**) *CXCR4*, *DLC1*, and *MAP1LC3C* are significantly enriched in pathways related to immune response and cancer in GSEA (FDR < 0.25, NP < 0.05).

As high expression of *CXCR4*, *DLC1*, and *MAP1LC3C* was significantly related to worse OS, we further analyzed the relationship of these genes with clinical features in GC, such as grade, clinical stage, and TNM stage. Consistent with outcome of the survival analysis, *CXCR4* was significantly upregulated in GC patients with younger ages and worse tumor status, including the grade 3 (*p* = 0.0062) and M1 stage, than in patients with other tumor statuses (*p* = 0.0194; Figure 3A). Moreover, *DLC1* and *MAP1LC3C* also showed a higher expression in the grade 3 group than in other groups (*p* = 0.0006 and *p* = 0.0012; Figure 3B, 3C). *CXCR4* and *MAP1LC3C* also showed a higher expression in the younger patients (age ≤ 65, *p* < 0.01; Figure 3A, 3C).

### Signaling pathways related to the prognostic DE-ATGs (CXCR4, DLC1, and MAP1LC3C)

We performed Gene Set Enrichment Analysis (GSEA) using the prognostic DE-ATGs in the high and low expression groups in the TCGA GC cohort. The analysis indicated that *CXCR4*, *DLC1*, and *MAP1LC3C* were significantly (FDR < 0.25, NP < 0.05) enriched in pathways related to immune response and cancer. The intersecting immune-related pathways of the three genes included primary immunodeficiency and T cell receptor signaling pathway; and those of the intersecting cancer-related pathways included pathways in cancer and VEGF pathway (Figure 3D–3F).

### Correlation of prognostic DE-ATGs (CXCR4, DLC1, and MAP1LC3C) with TIME using CIBERSORT, TIMER, and ESTIMATE

**Figure 4:**
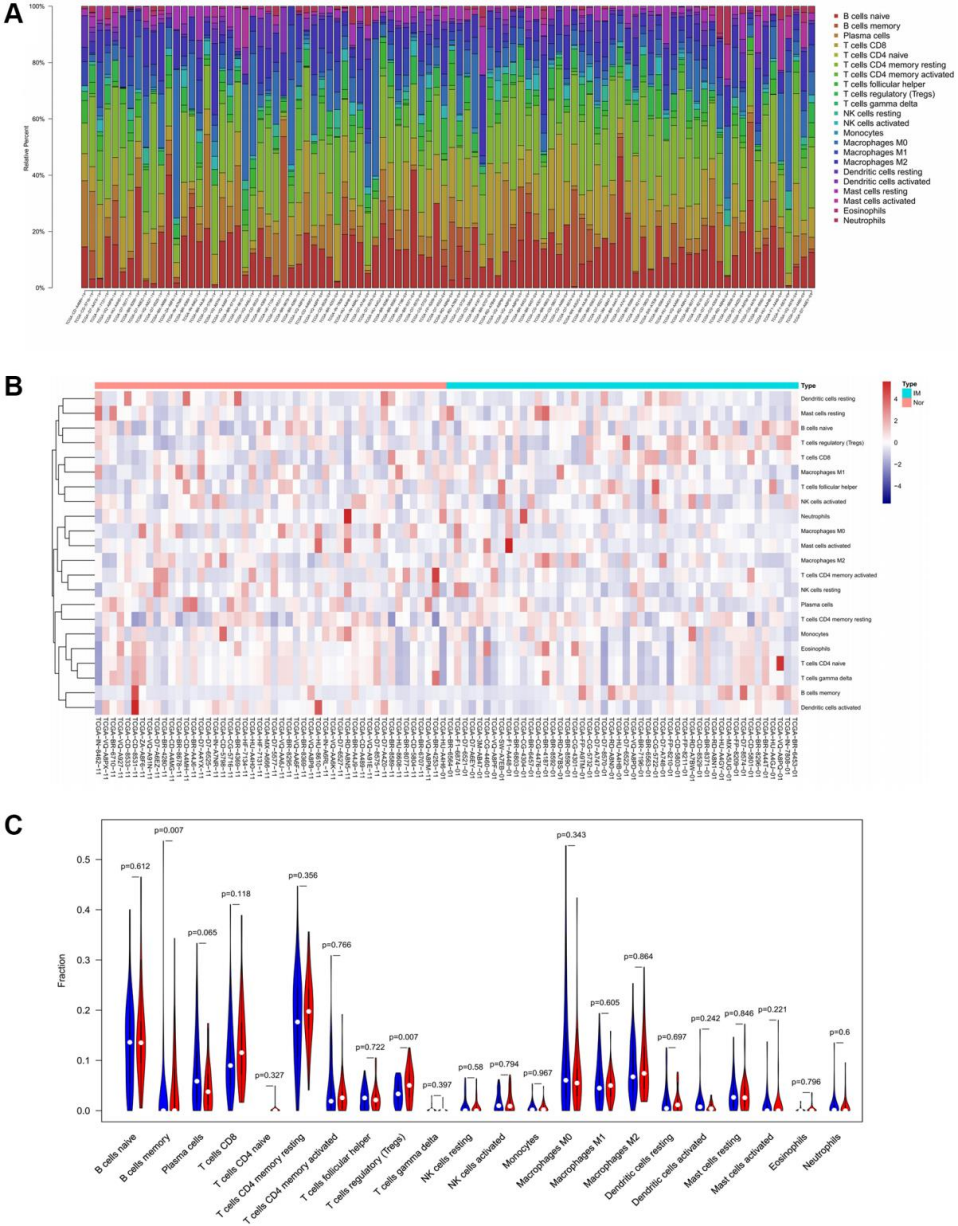
**Composition of 22 TIICs in the TCGA cohort with CIBERSORT *p* < 0.05 for all qualified samples.** (**A**) Fractions of 22 immune cells in qualified tumor samples (*n* = 96) in the TCGA. (**B** and **C**) Heatmap and violin plot comparing the immune cells between high- and low-CXCR4 expression groups.

**Figure 5:**
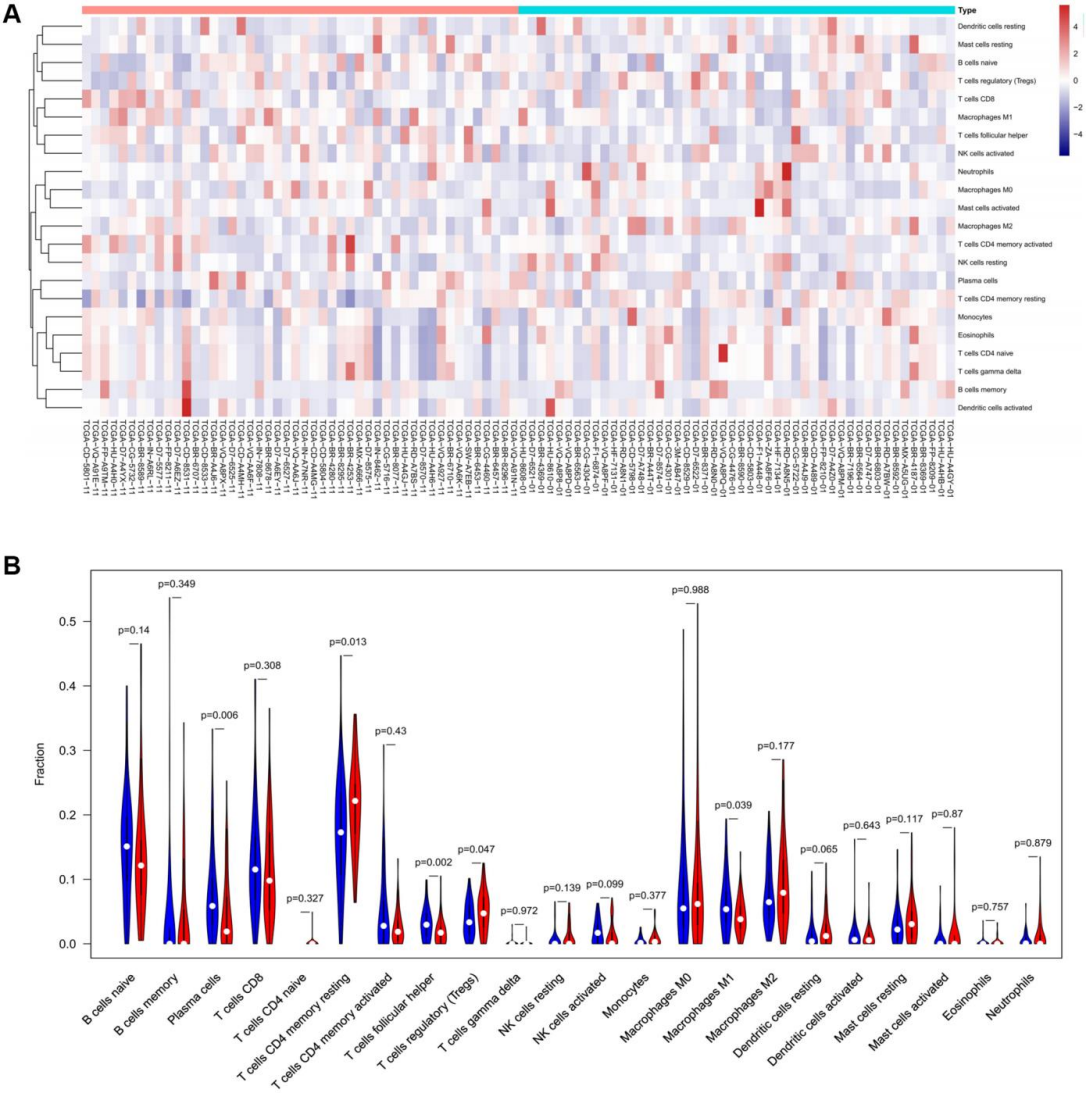
The distinct compositions of 22 TIICs in the high- and low-*DLC1* expression groups are shown using (**A**) heatmap and (**B**) violin plot and analyzed with CIBERSORT.

**Figure 6:**
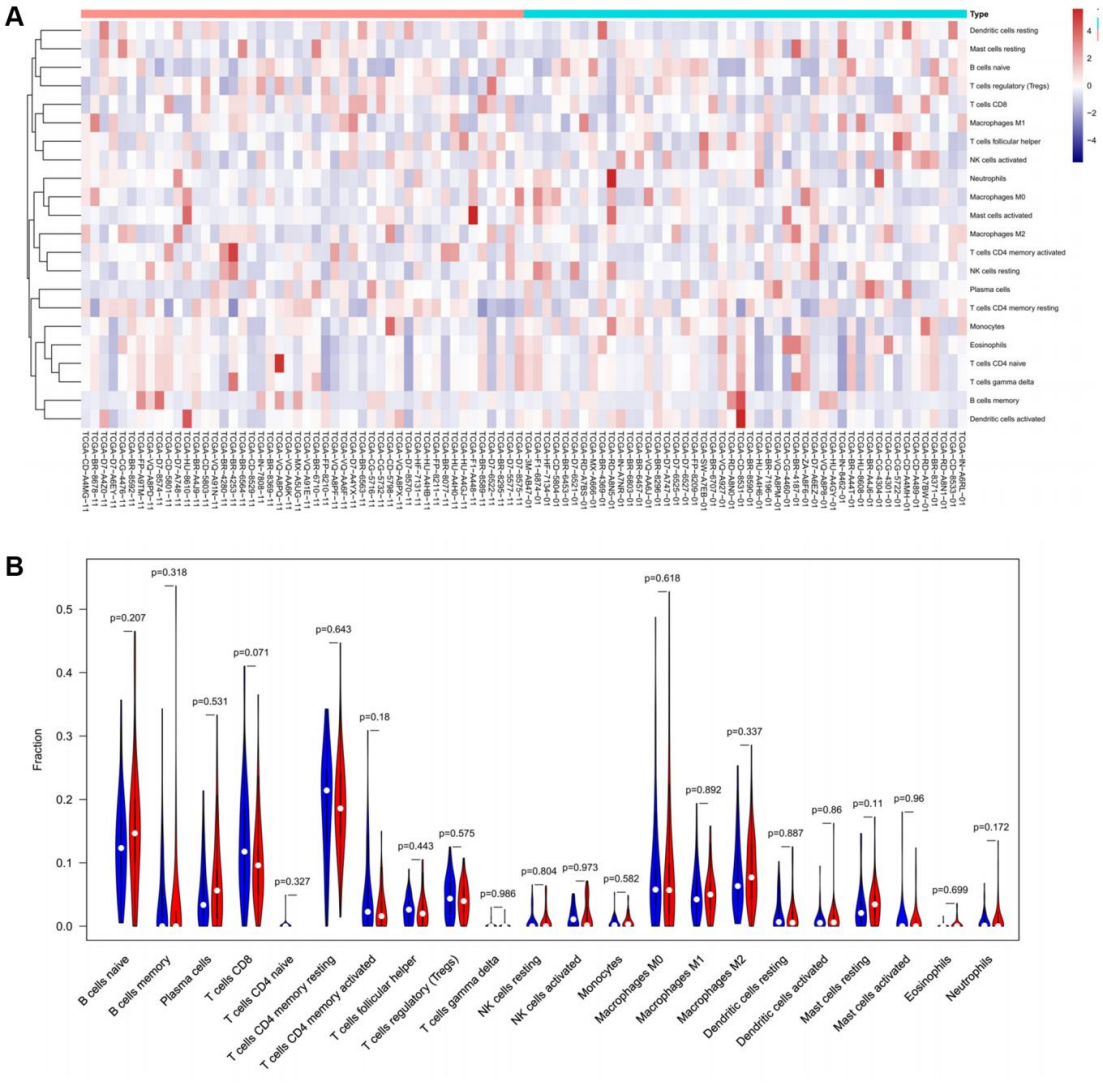
The distinct compositions of 22 TIICs in the high- and low-*MAP1LC3C* expression groups are shown using (**A**) heatmap and (**B**) violin plot and analyzed with CIBERSORT.

To verify the correlation between the prognostic DE-ATGs and immunity, we first investigated the differences between 22 subpopulations of tumor-infiltrating immune cells (TIICs) in DE-ATGs high and low expression groups using the CIBERSORT Algorithm [14]. Filtering with CIBERSORT *p* < 0.05, we selected 96 GC samples for the subsequent analysis (Figure 4A). The analysis suggested that compared with proportion in *CXCR4* low expression group, high *CXCR4* expression group had higher proportions of regulatory T cells (Treg cells, *p* = 0.007), and relatively lower proportions of memory B cells (*p* = 0.007; Figure 4B, 4C). Further, compared with proportions in low *DLC1* expression group (Figure 5A, 5B), the proportion of resting memory CD4+ T cells (*p* = 0.013) and Treg cells (*p* = 0.047) was significantly higher (*p* = 0.029), and that of plasma cells (*p* = 0.006), follicular helper T cells (*p* = 0.002) and M1 macrophages (*p* = 0.039) was significantly lower in high *DLC1* expression group. However, no significant differences in the proportions of immune cells between *MAP1LC3C* high- and low-expression groups were observed (Figure 6A, 6B).

**Figure 7:**
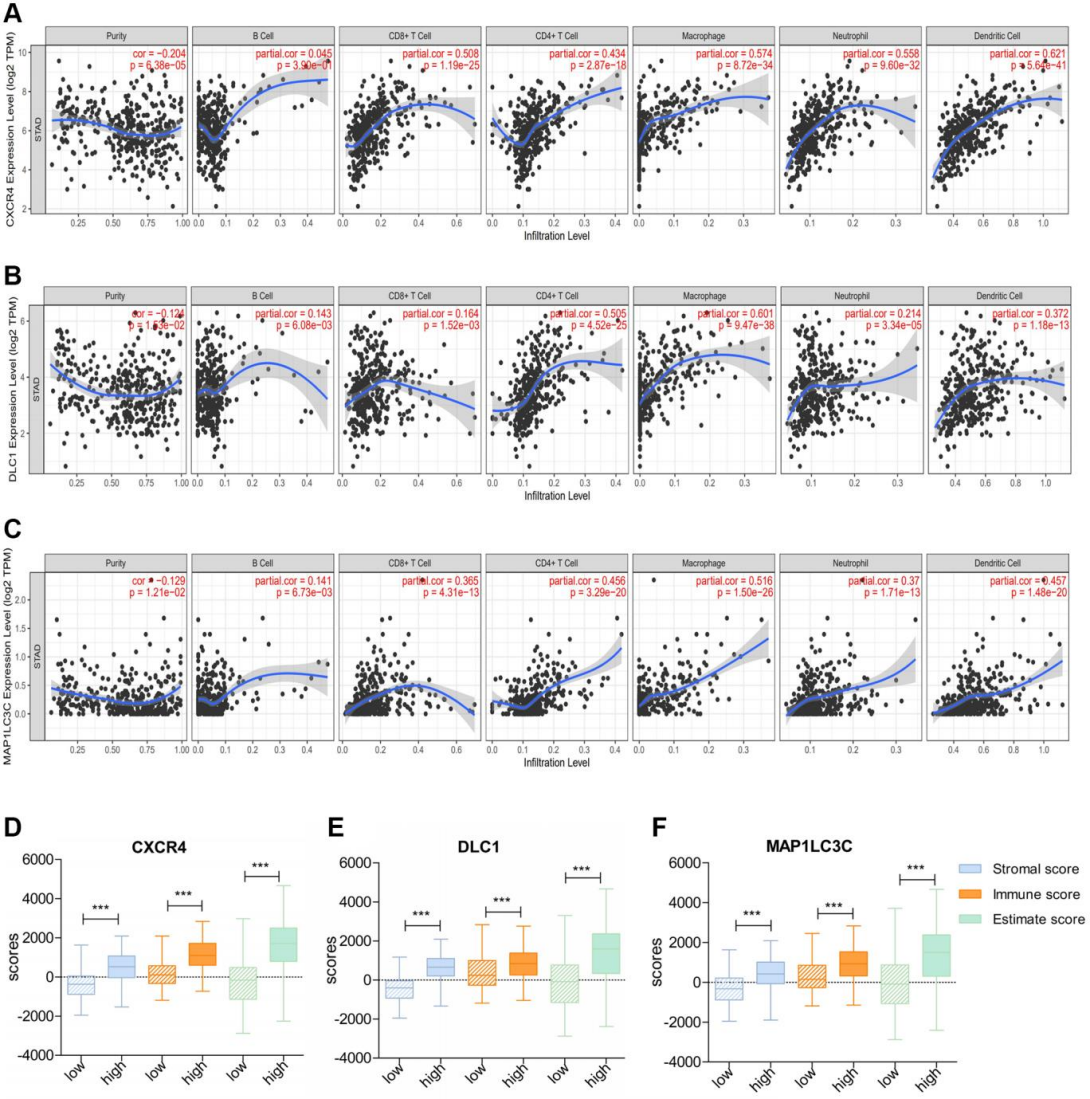
**Correlation of prognostic immune-related ATGs with characterization of the tumor immune environment.** (**A**–**C**) Relationship of *CXCR4*/*DLC1*/*MAP1LC3C* with B cells, CD8+ T cells, CD4+ T cells, macrophages, neutrophils, and dendritic cells using TIMER. (**D**–**F**) Distribution of ESTIMATE scores, immune scores, and stromal scores between high- and low-*CXCR4*/*DLC1*/*MAP1LC3C*-expression groups.

Next, we checked the correlation of DE-ATGs with TIICs using TIMER. The analysis showed that expression signature of *CXCR4* and *MAP1LC3C* had significant positive association with CD8+ T cells infiltration, CD4+ T cells infiltration, macrophage infiltration, neutrophil infiltration, and dendritic cells infiltration (Correlation coefficient >0.3; *p* < 0.05; Figure 7A, 7C). Moreover, the expression signature of *DLC1* was significantly positively correlated with CD4+ T cells infiltration, macrophage infiltration, and dendritic cells infiltration (Correlation coefficient > 0.3; *p* < 0.05; Figure 7B).

Consistent with above results, patients with high expression of all three DE-ATGs (*CXCR4, DLC1*, and *MAP1LC3C*) had a higher stromal score, immune score, and estimate score than those with low expression; this suggested that high-expression samples were infiltrated with more immune cells than low-expression samples (*p* < 0.05; Figure 7D–7F).

### Correlation of prognostic DE-ATGs (CXCR4, DLC1, and MAP1LC3C) with immune checkpoint inhibitor genes or surface molecules of immune cells

**Figure 8:**
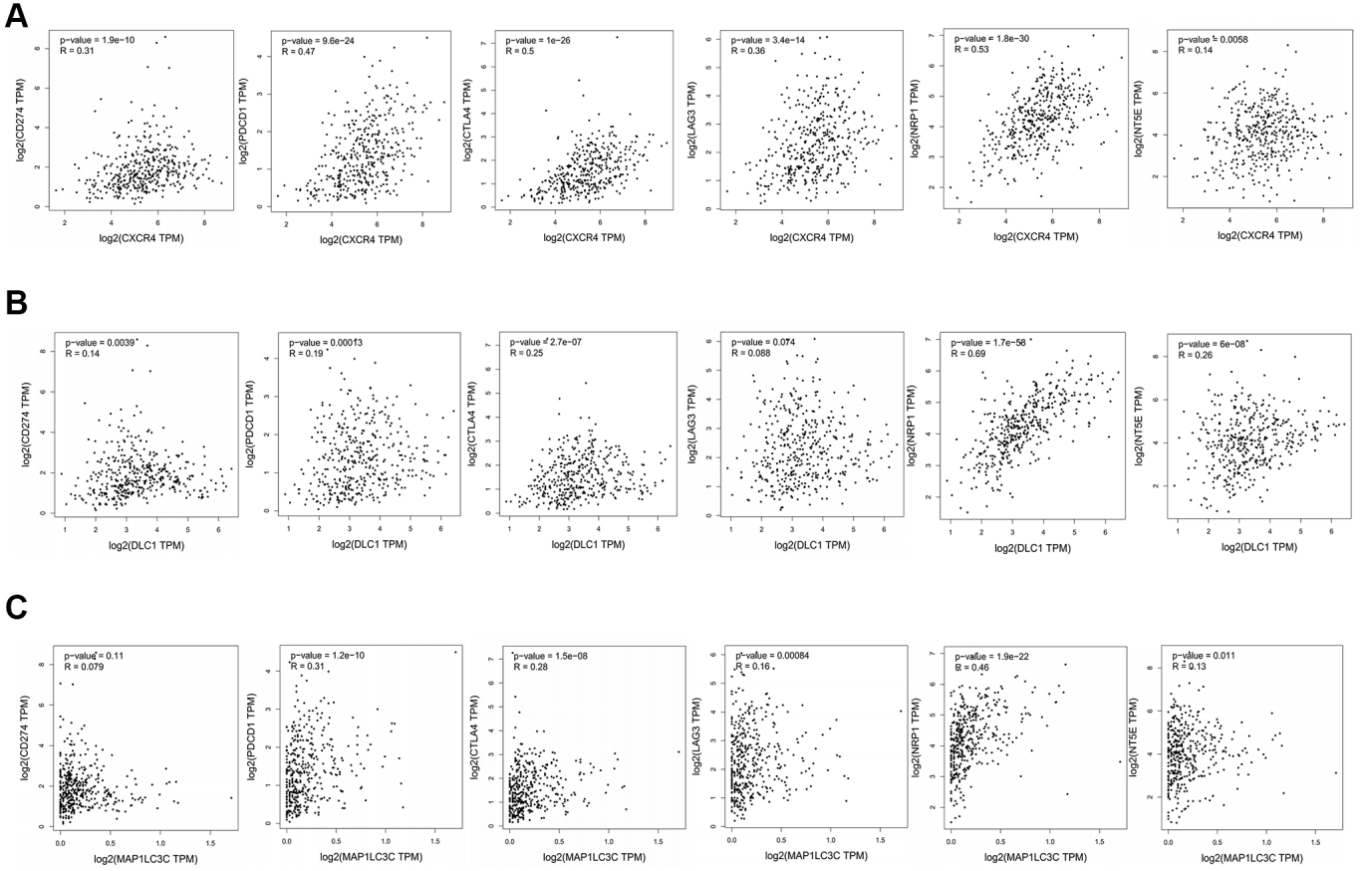
**Association between *CXCR4*/*DLC1*/*MAP1LC3C* and crucial immune checkpoint genes or surface molecules.** Analyses of association of ICB-related genes or surface molecules (*CD274*/*PDCD1*/*CTLA4/LAG-3/NRP-1/CD73*) with (**A**) *CXCR4*, (**B**) *DLC1*, and (**C**) *MAP1LC3C*.

Further, we checked correlation between prognostic DE-ATGs and immune checkpoint inhibitor genes or surface molecules of immune cells, using GEPIA, to elucidate the function of prognostic DE-ATGs in the immune checkpoint blockade (ICB) therapy. The analysis indicated that expression of *CXCR4* was significantly positively correlated to expression of *CD274* (R = 0.31; *p* < 0.05), *PDCD1* (R = 0.47; *p* < 0.05), *CTLA4* (R = 0.62, *p* < 0.001), *LAG3*(R = 0.36; *p* < 0.05), *CSF1*(R = 0.53; *p* < 0.05) and *NT5E* (R = 0.14; *p* < 0.05)in GC (Figure 8A); and, expression of *DLC1* was significantly positively correlated to expression of *CD274* (R = 0.14; *p* < 0.05), *PDCD1* (R = 0.19; *p* < 0.05) , *CTLA4* (R = 0.25, *p* < 0.001), *CSF1* (R = 0.69; *p* < 0.05) and *NT5E* (R = 0.26; *p* < 0.05) in GC (Figure 8B). The expression of *MAP1LC3C* was significantly positively correlated to expression of *PDCD1* (R = 0.31; *p* < 0.05), *CTLA4* (R = 0.26, *p* < 0.001), *LAG3* (R = 0.16; *p* < 0.05), *CSF1* (R = 0.46; *p* < 0.05) and *NT5E* (R = 0.13; *p* < 0.05) in GC (Figure 8C). Therefore, *CXCR4, PDCD1* and *MAP1LC3C* may function as non-negligible factors in cancer immunity.

## DISCUSSION

GC is a common malignancy and a leading cause of cancer-related mortality worldwide [1]. Moreover, despite the encouraging advancements made in cancer immunotherapy targeting the TME, only few patients with GC have achieved satisfactory therapeutic effects. The factors contributing to the poor curative effect in GC include tumor recurrence, metastasis, insensitivity to immunotherapy, heterogeneous molecular characterization, and poor selection of target genes [15, 16]. Therefore, it is important to develop accurate and powerful molecular biomarkers to elucidate novel effects of immunotherapy in GC.

**Figure 9:**
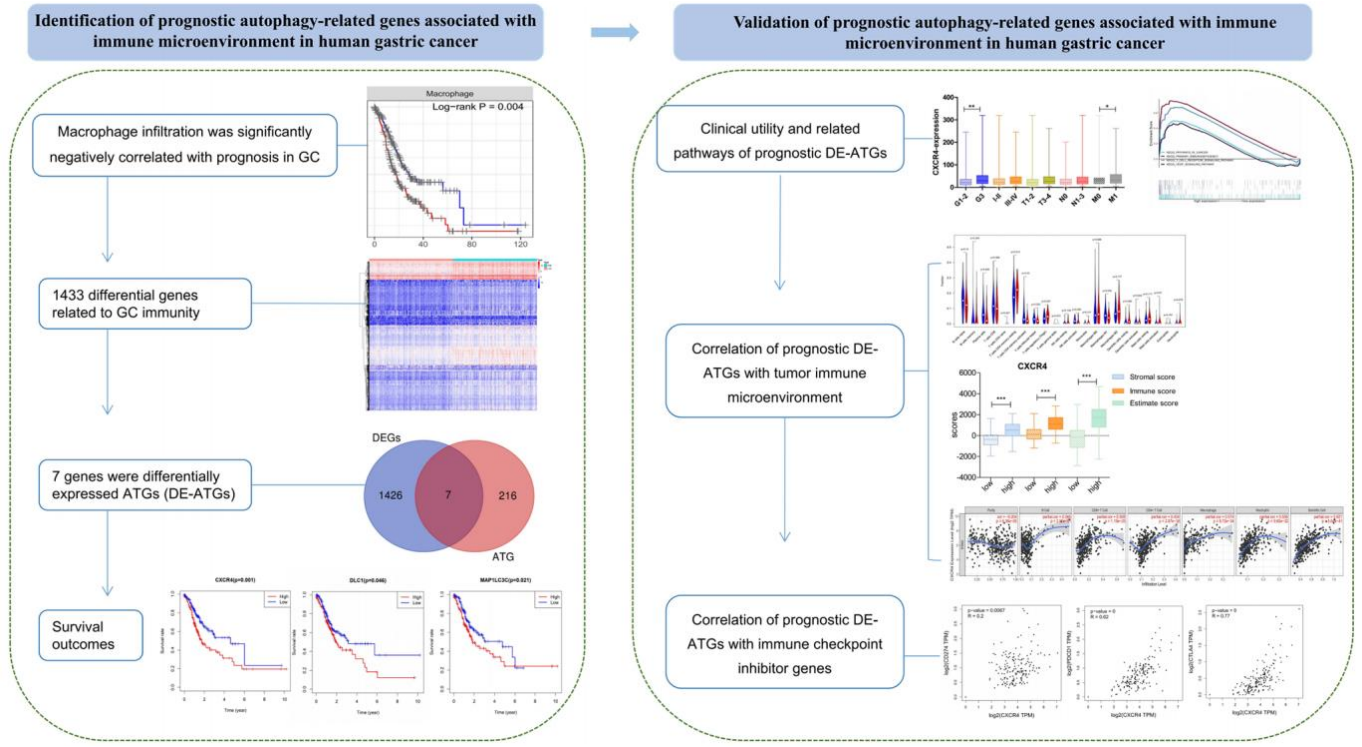
**The workflow of the study.** Construction and validation of the prognostic autophagy-related genes.

Autophagy is shown to play a key role in physiological and pathological processes. Moreover, dysregulated expression of ATGs influences tumorigenesis, progression, and therapeutic resistance in multiple cancers, including GC [9, 17, 18]. Therefore, DE-ATGs can be employed as new prognostic indicators and prospective therapeutic targets. Moreover, the use of new therapeutic avenues targeting autophagy may enhance the antitumor effects of immunotherapy, and improve the treatment outcome [8, 19]. In the present study, we found that macrophages that function as a bridge between autophagy and immunity [6, 7] were significantly negatively correlated with prognosis in GC. Further, three genes—*CXCR4*, *DLC1*, and *MAP1LC3C*—were identified as prognostic DE-ATGs that were associated with immune conditions via microphages (Figure 9).

Of the three prognostic DE-ATGs, *CXCR4* was found to be significantly upregulated and suggested poor prognosis in many cancer types, including GC [20, 21]. *CXCR4* performs important functions in regulating tumor growth, proliferation, metastasis, autophagy, and immune responses in cancers [22]. Studies have focused on the *CXCL12-CXCR4* chemokine axis due to its effects on macrophage recruitment and polarization, and immune cell migration [23, 24]. The effects of AMD3100, a *CXCR4* antagonist, in combination with PD-L1 and PD-1 inhibition have been tested as an ICB therapy in pre-clinical models [25–27]. Some of our findings are consistent with these previous bioinformatics or experimental observations that can be mutually complementary and verified [28–30]. But these studies are not comprehensive, and on top of that, we further checked the correlation between CXCR4 and surface markers of immune cells to elucidate the interaction between the CXCR4 and the potential related immune cells that may help improve strategies for *CXCR4* blockade in combinatorial immune-based therapies.

For *DLC1*, which is known to function as a GTPase-activating protein for Rho family members, plays an important role in cancer development and progression [31, 32]. In various cancer types, *DLC1* was identified as a potential tumor suppressor, however, the effects of *DLC1* do not work in only one direction [33]. The expression of *DLC1* has been reported to be reduced in gastric cancer, but the sample size in these studies was small [34, 35]. Regardless of the clear role of *DLC1* or its underlying mechanism remains elusive. The present study further clarified the prognostic value of *DLC1* and analyzed its interaction with the immune microenvironment.

Few existing studies have shown that the human Atg8-family protein-*MAP1LC3C* may be essential in particular biological responses, including cell autophagy, motility and invasion [36, 37]. Some bioinformatics analyses have revealed the potential value of *MAP1LC3C* in cancer [38, 39], but its role and mechanism in GC has seldom been discussed systematically up to now. The present study filled some gaps in the existing research, which identified higher expression of *MAP1LC3C* to be significantly associated with poorer prognoses and more advanced tumor grade 3. Moreover, the expression signature of *MAP1LC3C* had significant positive association with CD8+ T cells infiltration, CD4+ T cells infiltration, macrophage infiltration, neutrophil infiltration, and dendritic cells infiltration. Therefore, studying *MAP1LC3C* may provide newer insights for the prognosis and design of immune-based therapies in gastric cancer.

GSEA indicated that these prognostic genes were strongly associated with oncogenic and immune-related pathways. Then we found that all three prognostic DE-ATGs were closely associated with immune condition, both in terms of immune cells and immune scores. Various immune cells play different role in the immunosuppression and tumor progression, which are also related to prognosis of GC. Infiltration of some immune cells in tumors, such as CD8+ T cells, CD4+ T cells and NK cells, usually portends a more favorable prognosis. In contrast, tumors infiltrated by neutrophils and macrophages generally tend to have a poor prognosis [40]. Effector CD8+ T cells could produce a variety of chemokines to regulate tumor growth and development [41, 42]. As a major component of the TME, CD4+ T cells may attack tumor cells directly through cytolytic responses or indirectly through regulating other lymphocytes, such as strengthening B cell and cytotoxic T cells (CTLs) responses [43, 44]. Studies showed that N2 neutrophils inhibited the activity of anti-tumor CD8+ T cells by producing iNOS in breast cancer [45, 46]. DCs are also associated with immunosuppression and tumor progression via the initiation of CD8+ and CD4+ T cells [47]. However, the significant heterogeneity in the location and density of immune cells may affect their prognostic assessment [40]. As surface marker for CD8+ T cells, CD4+ T cells, and NK cells, LAG-3 can suppress their proliferation and effector activity [47–49]. NRP-1 is expressed in T cells and dendritic cells (DCs) and is associated with their activity and interaction [50]. CD73 (NT5E) is a potential surface marker for macrophage [51]. ICB immunotherapy (*CD274*, *CTLA4*, and *PDCD1*) is also being integrated into first-line therapy and gaining greater importance [3, 52, 53]. As the significant correlation between DE-ATGs and immune cells or surface makers of immune cells, we hypothesized DE-ATGs may connect autophagy with immunity via different surface markers (Supplementary Figure 1).

The bioinformatics-based studies on ATGs have focused on prognostic role of ATGs in GC [28, 54]. In contrast, the present study evaluated the link between autophagy and cancer immunity via macrophages to screen immune-related prognostic ATGs, which has not been elucidated previously. We believe that the identified immune-related prognostic ATGs may aid in developing combined immunotherapy and predict the response rate to immunotherapy in GC patients. However, the study has a few limitations. First, the study was heavily dependent on the publicly available datasets that provide less information about the ICB therapy administered in patients. Second, the function and significance of ATGs identified in the study remained to be elucidated; but we will attempt to clarify their role in future experimental *in vitro* and *in vivo* validation studies.

In summary, the present study provides immune-related ATGs that predict the OS in patients with GC. We also explored the clinical utility and verified the comprehensive landscape of TIME for these prognostic immune-related ATGs. We believe that the outcome of the present study may aid in better understanding of the ATGs and their interaction with the immune microenvironment, which would allow the development of novel inhibitors, personalized treatment, and immunotherapy in GC.

## MATERIALS AND METHODS

### Data sources

Level 3 gene expression profiles and clinical characteristics of patients diagnosed with GC were obtained from the TCGA database (https://cancergenome.nih.gov/, Supplementary Table 1, till October 18, 2020). The expression data were initially normalized using Trimmed mean of M values (TMM) with the edgeR Bioconductor package. The Human Autophagy Database (HADb, http://www.autophagy.lu/) was assessed to obtain the 232 ATGs. Immune scores and stromal scores were determined using ESTIMATE (https://bioinformatics.mdanderson.org/estimate/) data.

### Identification of DEGs

A correlation between the levels of TIICs and survival in patients with GC was established using TIMER (https://cistrome.shinyapps.io/timer/, till October 18, 2020). Further, we distributed patients with GC into two groups in accordance with the macrophage infiltration levels that were negatively correlated with patients’ OS. Adjusted *P*-value (adj.P) < 0.01 and |fold change (FC)| > 1.0 were applied to identify DEGs between the two groups using edgeR package in R version 4.0.3. Heatmap and volcano plots of DEGs were generated using pheatmap2 package in R software. DE-ATGs were obtained by intersection of the DEGs and ATGs, and visualized using Venn diagrams.

### Survival analysis and prognostic DE-ATGs

To identify potential prognostic DE-ATGs, we distributed patients with GC into high- and low-expression groups in accordance with the median expression levels of DE-ATGs. Further, log-rank test was assessed using R software to estimate the correlation between expression levels of DE-ATGs and OS, and the results were visualized using Kaplan-Meier (K-M) survival curve. Prognostic independence was validated using multivariate Cox Proportional Hazards model of the DE-ATGs and other clinic-pathological factors.

### Gene set enrichment analysis (GSEA)

GSEA (http://www.broadinstitute.org/gsea/) was performed to assess related pathways and molecular mechanisms in patients with GC. For each analysis, we performed 1,000 times gene set permutations to obtain a normalized enrichment score (NES) and an enrichment score (ES). Enriched gene sets with a normalized *P* (NP) < 0.01 and false discovery rate (FDR) < 0.25 were considered statistically significant.

### Correlation of prognostic DE-ATGs with characterization of TIME

The abundance of 22 types of TIICs—naive B cells, memory B cells, plasma cells, CD8+ T cells, naive CD4+ T cells, resting memory CD4+ T cells, activated memory CD4+ T cells, follicular helper T cells, Treg cells, gamma delta T cells, resting NK cells, activated NK cells, monocytes, M0 macrophages, M1 macrophages, M2 macrophages, resting dendritic cells, activated dendritic cells, resting mast cells, activated mast cells, eosinophils, and neutrophils—was determined using the CIBERSORT algorithm. CIBERSORT uses a previously reported statistical method to quantify the infiltrated immune cell composition fractions on the basis of gene signature matrix [14]. Only 96 GC samples with CIBERSORT *p* < 0.05 were selected for the subsequent analysis. Further, these 96 GC samples were distributed into high- and low-expression groups in accordance with the median expression levels of prognostic DE-ATGs. The Wilcoxon test was performed to analyze differential infiltrations of the 22 TIICs in the two groups. Correlations between different immune cells were tested by R package corrplot.

### Correlation between expression levels of prognostic DE-ATGs and abundance of TIICs

We selected ICB-related genes or surface molecules for immunotherapy: programmed death ligand 1 (PD-L1 or CD274), programmed death 1 (PD-1 or PDCD1), cytotoxic T-lymphocyte antigen 4 (CTLA-4), LAG-3, NRP-1, and CD73(NT5E). Further, we analyzed their expression levels between low- and high-expression prognostic DE-ATGs groups using Gene Expression Profiling Interactive Analysis (GEPIA, http://gepia.cancer-pku.cn/).

### Statistical analysis

All analyses were performed using R software version 4.03. For all statistical tests, *p* < 0.05 was considered as statistically significant. The DEGs were evaluated with Wilcoxon test using R software. Wilcoxon test was performed to analyze the different expression levels of prognostic DE-ATGs in several subgroups of clinical features.

### Availability of data and materials

The datasets analyzed during the current study are publicly available from the following online databases: TCGA (https://tcga-data.nci.nih.gov/tcga/); Human Autophagy Database (HADb, http://www.autophagy.lu/); ESTIMATE (https://bioinformatics.mdanderson.org/estimate/); TIMER (https://cistrome.shinyapps.io/timer/); GEPIA (http://gepia.cancer-pku.cn/).

### Ethics approval and consent to participate

The databases are publicly available and open to access, so this study did not need the approval from the ethics committee.

## Supplementary Materials

Supplementary Figure 1

Supplementary Table 1
