# Supplementary material for: Identification and validation of prognostic autophagy-related genes associated with immune microenvironment in human gastric cancer
Source: Aging (Albany NY). 2022 Sep 28;14(18):7617–34. doi: 10.18632/aging.204313 (PMC9550254; doi:10.18632/aging.204313)
Supplement: Supplementary Figure 1 [file aging-14-204313-s001.pdf]

SUPPLEMENTARY FIGURE

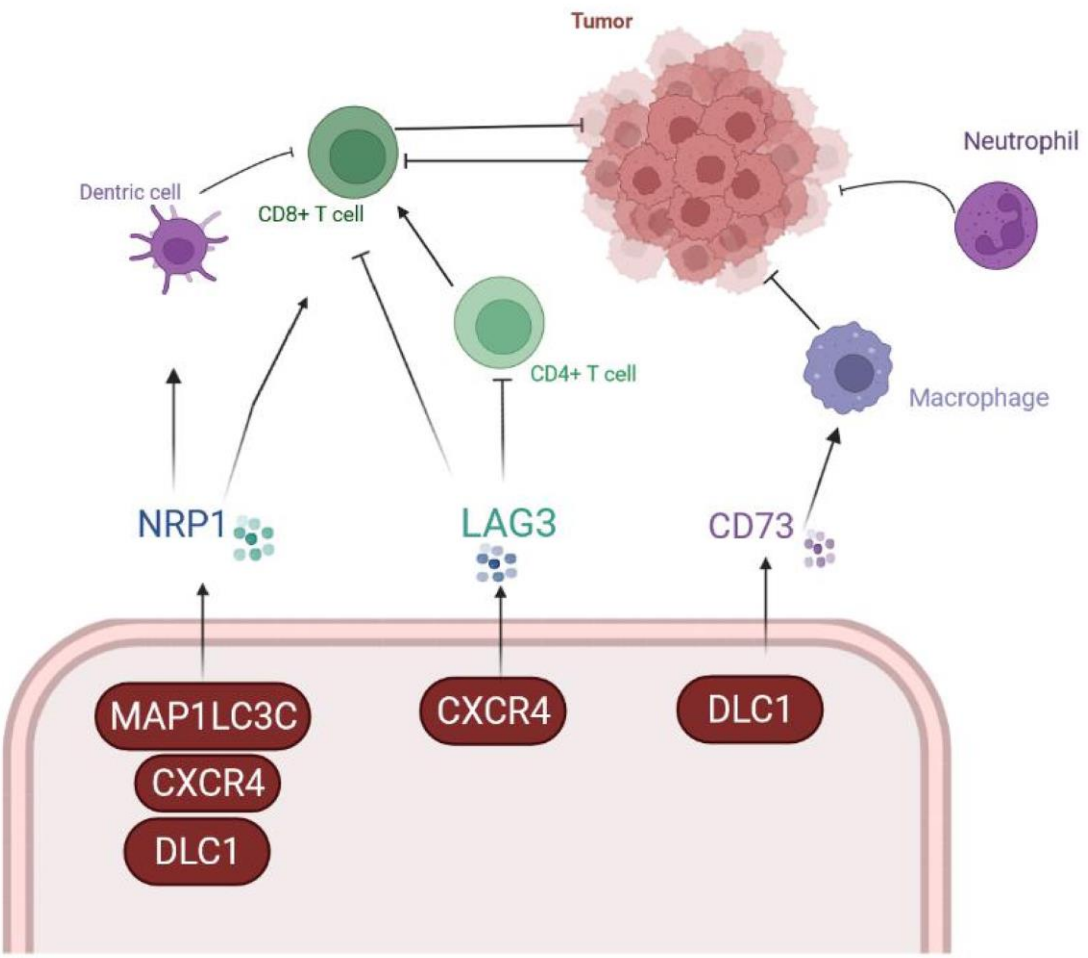

Supplementary Figure 1. The interactions between DE-ATGs and immune cells or surface markers of immune cells.
