# Supplementary material for: Identification and validation of prognostic autophagy-related genes associated with immune microenvironment in human gastric cancer
Source: Aging (Albany NY). 2022 Sep 28;14(18):7617–34. doi: 10.18632/aging.204313 (PMC9550254; doi:10.18632/aging.204313)
Supplement: Supplementary Table 1 [file aging-14-204313-s002.pdf]

SUPPLEMENTARY TABLE

Supplementary Table 1. Clinical and pathological characteristics of GC patients.

Characteristics	Count	Percent (%)
Size		
	366	100
Sex		
Male	232	63.40
Female	134	36.60
Size		
Age (years)		
≥60	247	67.49
<60	119	32.51
Stage		
I–II	160	43.72
III–IV	183	50.00
unknown	23	6.28
T-stage		
T1-T2	97	26.50
T3-T4	261	71.31
Tx	8	2.19
N-stage		
N0	108	29.51
N1-3	240	65.57
Nx	18	4.92
M-stage		
M0	322	87.98
M1	44	12.02
Grade		
G1&G2	144	39.34
G3	213	58.20
Gx	9	2.46
